# Changing Trends in Carotid Revascularization in Australia: A Nationwide Study Over 30 Years

**DOI:** 10.1111/ans.70159

**Published:** 2025-05-12

**Authors:** David F. Sun, Kevin Tian, Amanda Seneviratne, Oh Sung Choy, Daphne Wang, Vimalin Vedanayagam, Michelle T. Sun, Christopher X. Wong

**Affiliations:** ^1^ The Department of Vascular and Endovascular Surgery Townsville University Hospital Townsville Queensland Australia; ^2^ The Department of Plastics and Reconstructive Surgery Princess Alexandra Hospital Brisbane Queensland Australia; ^3^ Department of Ophthalmology Royal Adelaide Hospital and University of Adelaide Adelaide Australia; ^4^ Department of Cardiology Royal Adelaide Hospital and University of Adelaide Adelaide Australia

**Keywords:** carotid artery disease, carotid artery endarterectomy, carotid artery stenting

## Abstract

**Background:**

Carotid artery stenosis is one of the causes of acute ischaemic stroke. Carotid endarterectomy (CEA) and carotid artery stenting (CAS) are the main procedural treatment options. This study investigates the changing trends in carotid revascularisation in Australia over the last 30 years.

**Methods:**

Two population level datasets were used, the Australian Institute of Health and Welfare (AIHW) and Medicare Australia. Patients who had either CEA or CAS procedures between 1993 and 2024 were identified, and procedural trends were analyzed over a 30‐year period.

**Results:**

Data from AIHW was available from 2000 to 2021, over which 66 983 patients underwent carotid revascularisation (58 932 CEA and 8051 CAS). There was a 47.4% relative decrease in the absolute number of CEA procedures over the study period (3702–1948). There was a relative 23.1% increase in CAS procedures over this period (524–645). Data from the Medicare dataset was available from 1993 until 2024, over which 41 860 patients had carotid revascularisation (38 118 CEA and 3742 CAS). There was a relative 51.0% decrease in the absolute number of CEA procedures (1733–849). There was a 1.6% relative decrease in the absolute number of CAS procedures over the study period (187–184).

**Conclusion:**

The number of carotid revascularisation procedures performed has steadily decreased over the last 30 years. CEA procedures have declined to a greater extent compared to CAS procedures, though CEA procedures remain significantly more common. These trends likely reflect evolving medical therapy for carotid artery disease.

## 1 Introduction

Carotid artery stenosis is one of the leading causes of acute ischaemic stroke [1]. Symptomatic carotid stenosis is defined as one that is associated with amaurosis fugax, transient ischaemic attack, or stroke within a 6‐month time period [1]. Current recommendations from the European Society for Vascular Surgery (ESVS) state that patients with symptomatic carotid disease (50%–99% stenosis) should undergo carotid revascularisation within 14 days of symptom onset [2]. Conversely, the same guideline suggests that at least some individuals with asymptomatic carotid disease who are deemed to have a low stroke risk may be managed with best medical therapy (BMT), which includes an antiplatelet, high‐dose statin, and comprehensive risk factor modification [2].

Carotid artery stenting (CAS) is an alternative option for patients with symptomatic carotid stenosis, ranging from 50% to 99%, who are high risk for carotid endarterectomy (CEA) or have contra‐indications to neck surgery such as prior neck radiation or presence of tracheostomy [2, 3]. Retrospective and prospective series show that CEA has a lower perioperative stroke and mortality rate compared to CAS, but similar long‐term outcomes [2, 4, 5]. With the development of endovascular techniques, CAS has become a popular revascularisation modality in Australia, especially in patients not fit to undergo a CEA operation.

The role of surgical intervention for treatment of asymptomatic carotid stenosis has been a subject of ongoing debate. Nevertheless, advancements in modern BMT over the past 20–30 years have demonstrated comparable reductions in major stroke rates, as well as combined stroke and mortality rates, when compared to carotid interventions for asymptomatic carotid disease [6]. The aim of this study is to characterize the changing trends in carotid revascularisation treatments (CEA and CAS) using both public and private hospital data from two prospectively collected, nationwide Australian datasets: the Australian Institute of Health and Welfare (AIHW) and Medicare Australia (Medicare).

## 2 Methods

Prospectively collected de‐identified data on CEA and CAS procedures performed in Australia over a 30‐year period between 1993 and 2024 was collected and retrospectively analyzed. Two population level datasets were used, the AIHW and the Medicare Australia database of statistics on item numbers (Medicare).

This study adhered to the principles outlined in the Declaration of Helsinki. Institutional ethics review board approval was not required as the data used is available in a public repository, freely accessible, and nonidentifiable. Participant consent was not obtained due to the public and de‐identified nature of the data.

### 2.1 AIHW Dataset

The AIHW data represents nationwide statistics combining procedures performed both in public and private hospitals. Procedural data from the AIHW database is derived from the National Hospital Morbidity Database which is contributed to by each of the state and territory health authorities in Australia. The data are derived directly from electronic summary records for episodes of care in Australia. The proportion of missing data is negligible, representing 0.004% of cases per year [7]. The database contains information regarding the type of procedure, year of procedure, gender of patient, age group, and whether the procedure required same day or overnight admission. Procedure type is classified according to the second edition of the International Statistical Classification of Diseases and Related Health Problems, 10th Revision, Australian Modification (ICD‐10‐AM) and the 3rd to 6th editions of Australian Classification of Health Interventions (ACHI) [8].

For the present analysis, a database search was performed by two independent authors (D.F.S. and C.X.W.) for procedural data on CEA and CAS on available AIHW data cubes (number of procedures, year, age, and gender) over a 21‐year period between 2000 and 2021. The procedure codes for CEA included “33500‐00 + 32703‐00” and CAS “35307‐00 + 35307‐01.” Percutaneous transluminal angioplasty of single carotid artery single stent and multiple stents “35307‐00 + 35307‐01” were combined and classified under CAS treatment for carotid stenosis. Data on CEA procedures was available from 2000 to 2021, whereas data on CAS procedures was available from 2008 to 2021.

### 2.2 Medicare Australia Dataset

Procedural data from the Medicare database is derived from billing claims made for procedures. These procedures are typically performed in the private hospital sector but also include procedures on private patients in public hospitals. The Medicare database contains information about types of procedure according to billing codes, patient age and gender, and the number of procedures performed in each state.

For the present analysis, database searches were performed by two independent authors (D.F.S. and C.X.W.) from procedural data (with datapoints including number of procedures, year, age, and gender) in available Medicare dataset data cubes over a 30‐year period between 1994 and 2023. Procedure type was classified according to the Medicare coding system. The procedural code for CEA was 33500 + 32703, while the code 35307 was collected for CAS. Data on CEA procedures was available from 1993 to 2023, whereas data on CAS procedures was available from 2006 to 2023.

### 2.3 Statistical Analysis

Absolute procedural numbers are first reported, and the relative change procedural numbers over the study period were calculated.

To calculate procedural incidence rates, Australian estimated resident population data was first obtained using the online yearly estimates through the Australian Bureau of Statistics [9]. A population adjusted number of procedures performed per 100 000 persons was estimated to explore the yearly increases according to population size using the formula: (absolute numbers of procedures for year *X*/estimated resident population for year *X*) × 100 000. We then calculated the incidence rates for both CEA and CAS. The total number of CEA and CAS procedures was calculated in the following age groups (< 50, 50–59, 60–69, 70–79, and ≥ 80) and (≤ 54, 55–64, 65–74, 75–84, ≥ 85) respectively, and the procedures were standardized according to the age and gender structure of the Australian population in each relevant year.

Absolute incidence rates and the relative change in such incidence rates are first reported. Trends for the number of surgeries performed each year were subsequently assessed using negative binomial regression models. Negative binomial regression was further utilized to analyze if time trends in CEA and CAS varied according to age group. In such models, year (continuous), age group, gender, and the interaction between age group and year were included as predictors. Additionally, negative binomial regression was also used to assess if gender influenced procedural trends; in such models, year (continuous), age group, gender, and the interaction between gender and year were included as predictors. Trends are reported as incidence rate ratios with associated 95% confidence intervals (CIs).

All analyses were conducted using Stata (version 16) and statistical significance was set at *p* < 0.05, by two independent authors (D.F.S. and C.X.W.).

## 3 Results

### 3.1 AIHW Dataset

Over the entire 21‐year period from 2000 to 2021, a total of 66 983 individuals underwent carotid revascularisation procedures in the AIHW dataset (58 932 CEA and 8051 CAS procedures; Table 1). From 2000 to 2021, there was a 47.4% relative decrease in the absolute number of CEA procedures (from 3702 to 1948). In contrast, from 2008 to 2021 there was a relative 23.1% increase in the absolute number of CAS procedures (from 524 to 645). After age and gender standardization, there was a decrease in the incidence rate of CEA by 61.0% (from 19.5 CEA procedures per 100 000 people to 7.6 CEA procedures per 100 000; Table 1). In contrast, while there was some variation over the study period in CAS incidence rates (with a minimum of 1.8 CAS procedures per 100 000 in 2012 to a maximum of 2.9 CAS procedures per 100 000 in 2019), there was no relative change in CAS incidence at the beginning and the end of the 2008–2021 period (2.5 CAS procedures per 100 000; Table 1). Negative binomial regression analysis demonstrated a 5.1% annual decrease in CEA incidence rates over the 2000–2021 study period (incidence rate ratio 0.949, 95% CI 0.946–0.950). In contrast, there was a no significant annual change in CAS incidence rates by negative binomial regression analysis from 2008 to 2021 (incidence rate ratio 1.010, 95% CI 0.999–1.020).

**TABLE 1:** Number and Incidence of CEA and CAS Procedures from 2000 to 2021 (AIHW).

Years	Absolute number		Incidence (per 100 000 population)	
	CEA	CAS	CEA	CAS
2000	3702		19.5	
2001	3808		19.8	
2002	3442		17.7	
2003	3143		15.9	
2004	2959		14.8	
2005	2771		13.7	
2006	2613		12.8	
2007	2637		12.7	
2008	2576	524	12.1	2.5
2009	2752	520	12.7	2.4
2010	2361	578	10.7	2.6
2011	2608	503	11.7	2.3
2012	2615	413	11.5	1.8
2013	2702	456	11.7	2.0
2014	2458	464	10.5	2.0
2015	2420	527	10.2	2.2
2016	2340	631	9.7	2.6
2017	2409	642	9.8	2.6
2018	2294	687	9.2	2.8
2019	2257	746	8.9	2.9
2020	2117	715	8.3	2.8
2021	1948	645	7.6	2.5
Relative increase/decrease (%)	−47.4	23.1	−61.0	0.0

**FIGURE 1:**
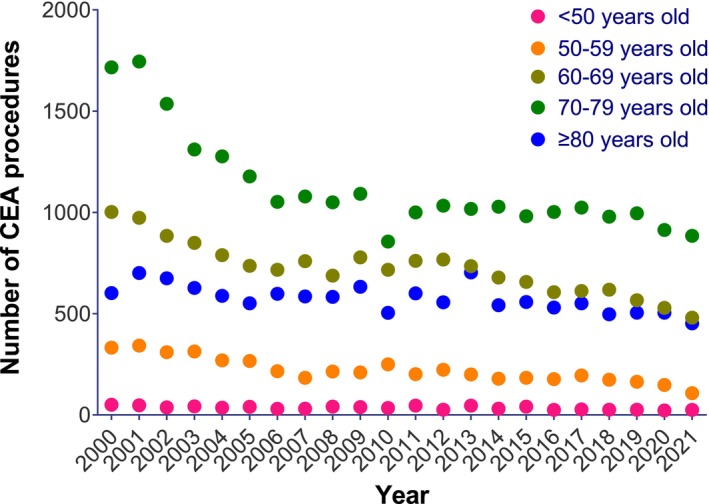
Absolute numbers of CEA by age group from 2000 to 2021 (AIHW).

**FIGURE 2:**
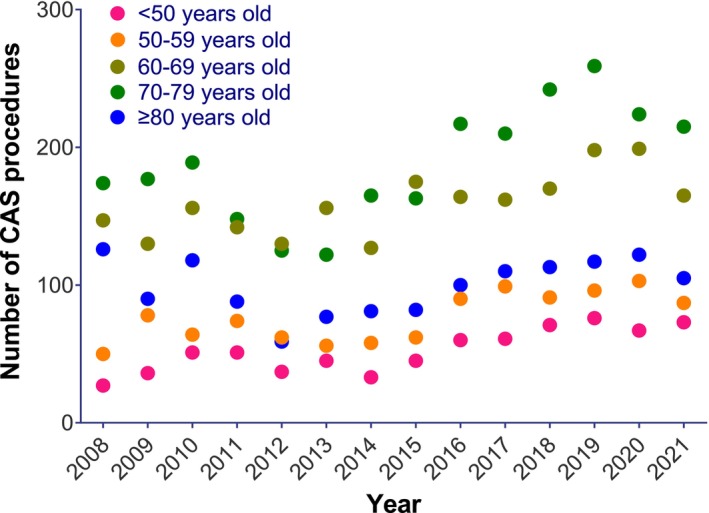
Absolute numbers of CAS by age group from 2000 to 2021 (AIHW).

When stratified by age group, CEA procedures appeared to demonstrate an annual decrease in procedural incidence rate across all age groups (*p* < 0.001 for all, Figure 1). Interaction testing was non‐significant, suggesting that the annual decrease in CEA incidence rates was similar across all age groups (Table 2). In contrast, CAS procedures appeared to demonstrate significant annual increases of 5.3% among the younger age group (< 50), but no significant annual change was observed among older age groups (Figure 2 and Table 2). Interaction testing suggested that diverging annual trends in CAS incidence rates by age group were statistically significant (*p* < 0.001, Table 2).

**TABLE 2:** Temporal trends in CEA and CAS procedures according to age group and gender (AIHW).

	Rate ratio	95% confidence interval	*p*	*p* for interaction
Age group (years)				
CEA				
< 50	0.964	0.953–0.975	< 0.001	0.41
50–59	0.947	0.940–0.953	< 0.001	
60–69	0.943	0.940–0.947	< 0.001	
70–79	0.948	0.943–0.952	< 0.001	
≥ 80	0.954	0.950–0.958	< 0.001	
CAS				
< 50	1.053	1.031–1.075	< 0.001	< 0.001
50–59	1.030	1.011–1.049	0.002	
60–69	1.001	0.990–1.013	0.885	
70–79	0.994	0.978–1.011	0.474	
≥ 80	0.984	0.964–1.004	0.117	
Gender				
CEA				
Male	0.950	0.947–0.953	< 0.001	0.10
Female	0.947	0.943–0.950	< 0.001	
CAS				
Male	1.006	0.993–1.018	0.363	0.38
Female	1.010	0.998–1.022	0.096	

Stratifying by gender demonstrated a 5.0% and 5.3% annual decrease in CEA for both males and females, respectively (Table 2). Interaction testing was not significant, suggesting the annual decrease was similar across both genders (*p* = 0.10). Stratifying by gender also demonstrated no significant annual change in CAS in both males and females, with no evidence of effect modification by gender (*p* = 0.38).

### 3.2 Medicare Dataset

Over the 30‐year period from 1994 to 2023, a total of 41 860 individuals underwent carotid revascularisation procedures in the Medicare dataset (38 118 CEA and 3742 CAS procedures; Table 3). From 1994 to 2023, there was a relative 51.0% decrease in the absolute number of CEA procedures (1733–849). In contrast, there was only a minor 1.6% relative decrease in the absolute number of CAS procedures (187–184) from 2006 to 2023. After age and gender‐standardization, there was a decrease in the incidence rate of CEA by 67.0% (9.7 per 100 000 people in 1994 to 3.2 per 100 000 people in 2023; Table 3). After age and gender‐standardization, there was a 22.2% decrease in CAS incidence rate (0.9 per 100 000 people in 2006 to 0.7 per 100 000 people in 2023). Negative binomial regression analysis demonstrated a 5.5% annual decrease in the incidence rates of CEA procedures (incidence rate ratio 0.945, CI 0.942–0.948). Similarly, negative binomial regression analysis showed a 4.2% annual decrease in the incidence rates of CAS procedures (incidence rate ratio 0.958, CI 0.947–0.968).

**TABLE 3:** Number and incidence of CEA and CAS procedures from 1994 to 2021 (Medicare).

Years	Absolute number		Incidence (per 100 000 population)	
	CEA	CAS	CEA	CAS
1994	1733		9.7	
1995	1966		11.0	
1996	1988		10.9	
1997	1951		10.6	
1998	1879		10.1	
1999	1727		9.2	
2000	1580		8.3	
2001	1474		7.6	
2002	1415		7.3	
2003	1357		6.9	
2004	1223		6.2	
2005	1152		5.7	
2006	1122	187	5.5	0.9
2007	1029	325	4.9	1.6
2008	1065	292	5.0	1.4
2009	1101	232	5.1	1.1
2010	1170	211	5.3	1.0
2011	1089	212	4.9	0.9
2012	1132	232	5.0	1.0
2013	1135	165	4.9	0.7
2014	1128	181	4.8	0.8
2015	1111	176	4.7	0.7
2016	1000	194	4.1	0.8
2017	1051	210	4.2	0.9
2018	1043	195	4.2	0.8
2019	917	207	3.6	0.8
2020	973	185	3.8	0.7
2021	918	186	3.6	0.7
2022	840	168	3.2	0.6
2023	849	184	3.2	0.7
Relative increase/decrease (%)	−51.0	−1.6	−67.0	−22.2

**FIGURE 3:**
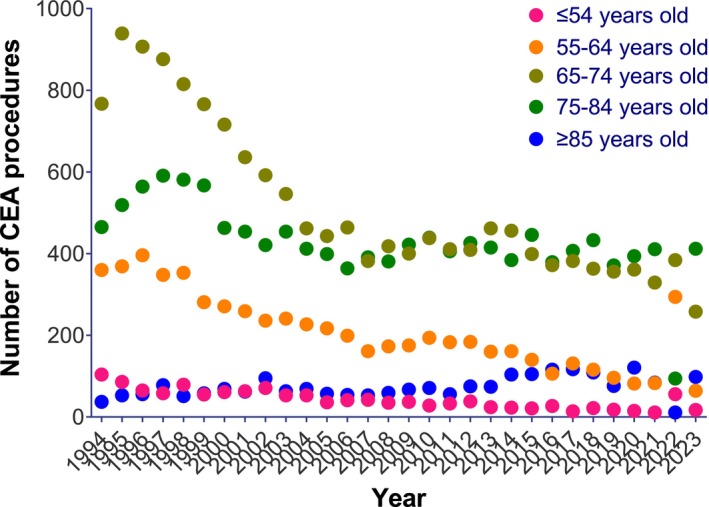
Absolute numbers of CEA by age group from 1994 to 2021 (Medicare).

**FIGURE 4:**
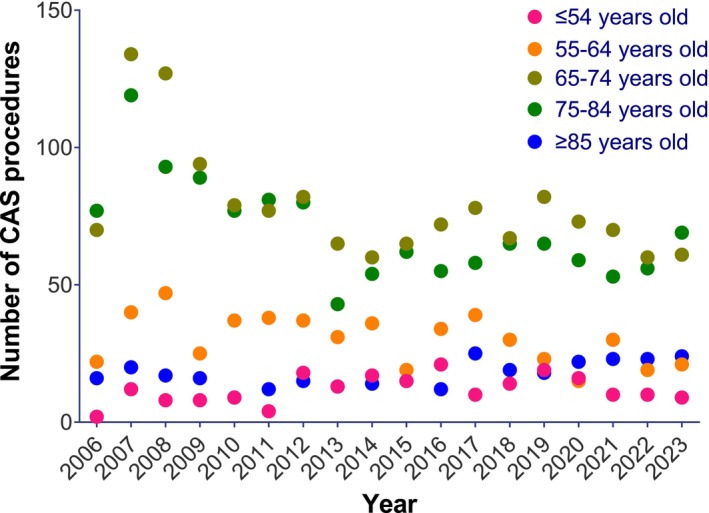
Absolute numbers of CAS by age group from 1994 to 2021 (Medicare).

When stratified by age group, there was an annual decrease in the incidence rates of CEA procedures in all age groups (*p* < 0.001 for all; Table 4). However, interaction testing suggested that the decrease was significantly different across age groups (*p* < 0.001), with the incidence rates of CEA procedures decreasing more rapidly among younger age groups (7.2% and 8.0% annual decreases among ≤ 54 and 55–65 years respectively; Figure 3) compared to older age groups (6.5%, 4.0%, and 2.0% annual decreases among 65–74, 75–84, and ≥ 85 years respectively; Table 4). CAS incidence rates also decreased in the three middle age groups (4.6%, 6.5%, and 5.8% annual decreases among 55–64, 65–74, and 75–84 years, respectively; Table 4 and Figure 4). In contrast, while the age groups of ≤ 54 and ≥ 85 showed a numerical increase in CAS incidence rates over time, these trends were not statistically significant (Table 4). Interaction testing for CAFS also produced a non‐significant result suggesting that incidence rates did not statistically differ across age groups (*p* = 0.09).

**TABLE 4:** Temporal trends in CEA and CAS procedures according to age group and gender (Medicare).

	Rate ratio	95% confidence interval	*p*	*p* for interaction
Age group (years)				
CEA				
≤ 54	0.928	0.920–0.935	< 0.001	< 0.001
55–64	0.920	0.916–0.925	< 0.001	
65–74	0.935	0.932–0.939	< 0.001	
75–84	0.960	0.955–0.965	< 0.001	
≥ 85	0.980	0.972–0.988	< 0.001	
CAS				
≤ 54	1.014	0.969–1.061	0.555	0.920
55–64	0.954	0.934–0.975	< 0.001	
65–74	0.935	0.921–0.949	< 0.001	
75–84	0.942	0.929–0.955	< 0.001	
≥ 85	1.014	0.976–1.055	0.468	
Gender				
CEA				
Male	0.948	0.944–0.953	< 0.001	0.012
Female	0.941	0.937–0.945	< 0.001	
CAS				
Male	0.962	0.947–0.978	< 0.001	0.663
Female	0.953	0.939–0.966	< 0.001	

Stratifying by gender showed a 5.2% and 5.9% decrease in CEA incidence rates in both males and females, respectively (incidence rate ratio 0.948 and 0.941, CI 0.944–0.953 and 0.937–0.945, respectively; Table 4). Interaction testing was significant, suggesting that the decrease in CEA incidence rates over time was greater in females (*p* = 0.012). Conversely, stratification by gender demonstrated similar decreases in CAS incidence rates in both males and females over time, with no significant interaction effect seen (*p* = 0.66).

## 4 Discussion

This retrospective study represents a nationwide dataset analyzing the trend in carotid revascularisation procedures in Australia over a 30‐year period. Our study demonstrated that over this time period, the number of CEA procedures has significantly decreased. On the other hand, the number of CAS procedures has remained relatively stable. Stroke represents a significant burden for Australian society. Recent data suggest that 6.7% of all adults in Australia suffered one or more conditions related to coronary, cerebrovascular, or other vascular disease in 2022 alone [9]. Carotid artery stenosis is associated with approximately 11.5% of ischaemic strokes [10]. A detailed understanding of the use of carotid revascularisation procedures to manage carotid artery disease is thus of great clinical and public health significance.

Over the last 30 years, the number of carotid interventions, specifically CEA procedures performed in Australia has significantly decreased, despite a steady increase in our ageing population, in whom carotid artery disease tends to be more common [11]. Notably, this decrease in CEA procedures was observed across all age groups, but particularly so at younger ages. While the reasons for this shift are likely multifactorial, one significant risk factor to consider is smoking. According to AIHW data, the proportion of people aged 14 and above who smoke daily has decreased from 24% in 1991 to 8.3% in 2023 [12]. This decline in smoking prevalence may have contributed to the reduced burden of carotid artery disease and, consequently, the decreasing need for invasive interventions [12]. Additionally, BMT with risk factor management for asymptomatic disease has demonstrated similar reductions in major stroke, combined stroke and mortality compared to CEA for asymptomatic disease [13, 14, 15]. A systematic review analyzing 17 studies with over 14 000 patients showed that in the pre‐2000s era CEA produced higher reductions in odds of stroke and mortality compared to BMT therapy [6]. In contrast, in the post 2000s era there was no significant difference between CEA and BMT therapies [6]. This may explain the shift towards a more conservative approach in managing asymptomatic carotid artery disease. The findings suggest that advances in BMT with intensive risk factor control have reduced the necessity for invasive procedures. As evidence‐based practice continues to shape clinical decision‐making, there has been a decline in the overall volume of open surgical interventions for carotid artery disease.

Our study also demonstrated that CAS was being performed slightly more frequently in younger individuals over this study period, but overall, the gross number of procedures performed is still vastly lower compared to the number of CEA. CAS was introduced as the “minimally invasive” alternative technique to treat carotid disease in the late 1990s and early 2000s in Australia for individuals who were unfit for CEA [10]. The CREST 1 trial, while showing a higher rate of perioperative stroke in CAS patients, showed that younger patients (< 70) had better outcomes with CAS [5]. More recent literature has shown that CAS is associated with a significantly higher risk of the combination of periprocedural death or stroke or ipsilateral stroke within 30 days of treatment compared with CEA [4, 16, 17]. CAS has shown up to a 31% increased risk of periprocedural death, MI, or stroke when compared to CEA [17]. These findings could explain the trends for CAS in our data, with the AIHW dataset showing only a small relative increase and the Medicare dataset showing very little change. This is especially true in patients who were above > 70 years old with a 2.23 times increased risk of periprocedural death or stroke when undergoing a CAS procedure, which supports the CREST 1 trial results [4]. Furthermore, this may explain the significant annual increases of CAS procedures of 5.3% only among young age groups < 50 in the AIHW dataset.

Our study demonstrated a decrease in carotid‐based interventions over a 30‐year period, a trend that is echoed in other healthcare systems. A recent 2021 study of the Australian Vascular Audit data between 2010 and 2017 reported a decrease of 23% and 37% for CEA and CAS respectively [16]. Our study showed a higher CEA procedural decrease rate of 47.4% and 51.0% for the AIHW and Medicare data respectively. However, our study confirmed a lower CAS procedure decrease rate in our Medicare data of 22.2%. This may be due to the data set extending over a longer time period and including data from a greater number of hospitals. A similar trend was observed in a UK‐based study displaying an almost 30% decline [18]. On the other hand, a Canadian study portrayed a 72% increase in CAS procedures over a 13‐year period, although both US‐ and Canadian‐based studies showed varying trends for CAS [19]. This demonstrates that, while CEA procedures appear to be consistently declining, the rates of CAS may vary across different health systems globally [19, 20, 21].

Our study confirms that CEA remains the favored procedure for carotid revascularisation in Australia. The study also demonstrates that the total number of CEA procedures has decreased likely due to advancements in BMT for asymptomatic individuals and intensive risk factor modification, though this could be confirmed in further studies with data on clinical indication for correlation. This landscape may continue to evolve as the ongoing CREST‐2 trial comparing the best method of stroke prevention in carotid artery stenosis may direct clinicians to follow a new trend [22]. As technology and techniques continue to develop for carotid revascularisation, we may see a shift in future trends requiring further study. Our findings on changing interventional practice have important implications for clinical and population health planning in Australia and beyond.

## 5 Limitations

Our analysis has several limitations that warrant recognition. Neither the AIHW nor the Medicare dataset recorded the indication for carotid revascularisation. Thus, we were unable to deduct the rates of asymptomatic versus symptomatic carotid intervention. While major strengths of the datasets are their comprehensiveness, nationwide nature, and extended timeframe, another limitation is the absence of individual patient‐level detail such as comorbidities. Additionally, the code 32703‐00 (AIHW) and 32703 (Medicare) did not separate those that had an endarterectomy versus those that did not, which could have overestimated or underestimated our final results. Another limitation relates to the reliance on diagnostic coding and the database lacking additional details such as hospital and state‐specific information (AIHW) which may have provided additional insight into our observed trends.

## 6 Conclusion

The number of carotid revascularisation procedures performed has steadily decreased over the last 30 years. CEA procedures have declined to a greater extent compared to CAS procedures, though CEA procedures remain significantly more common. These trends likely reflect evolving medical therapy for carotid artery disease.

## Author Contributions

**David F. Sun:** data curation, formal analysis, investigation, writing – original draft. **Kevin Tian:** writing – review and editing. **Amanda Seneviratne:** writing – review and editing. **Oh Sung Choy:** writing – review and editing. **Daphne Wang:** writing – review and editing. **Vimalin Vedanayagam:** writing – review and editing. **Michelle T. Sun:** data curation, formal analysis, methodology. **Christopher X. Wong:** conceptualization, data curation, formal analysis, investigation, methodology, supervision, writing – reviewing and editing.

## Conflicts of Interest

Dr. Wong reports that his institutions have received on his behalf lecture, travel, and/or research funding from Abbott, Bayer, Boehringer Ingelheim, Boston Scientific, Medtronic, Microport, Novartis, Servier, St Jude Medical, and Vifor Pharma.
